# Validation of SUSPEKT Score in Predicting One-month Mortality of Patients with Hemorrhagic Stroke; a Diagnostic Accuracy Study

**Published:** 2019-09-29

**Authors:** Hamid Kariman, Hamidreza Hatamabadi, Majid Shojaee, Farhad Asarzadegan, Simin Saljughi

**Affiliations:** 1 Emergency Department, Imam Hossein Hospital, Shahid Beheshti University of Medical Sciences, Tehran, Iran.; 2 Neurology Department, Imam Hossein Hospital, Shahid Beheshti University of Medical Sciences, Tehran, Iran.

**Keywords:** Intracranial hemorrhages, stroke, decision support techniques, prognosis, patient outcome assessment, mortality

## Abstract

**Introduction:**

Predicting the outcome of patients with intracranial hemorrhage (ICH) is the area of interest for in charge physicians as well as patients and their associates. This study aimed to evaluate the accuracy of SUSPEKT score in predicting one-month outcome of patients with hemorrhagic stroke.

**Methods:**

This prospective cross sectional study was conducted on > 18 years old patients with non-traumatic supra-tentorial ICH admitted to emergency department, from February 2017 to January 2018. SUSPEKT score was measured for each patient and its screening performance characteristics in prediction of one-month mortality were calculated.

**Results:**

169 cases with the mean age of 63.09± 15.45 (21 – 96) years were studied (56.8% male). After one month follow up 47 (27.8%) cases had died, 30 (17.7%) cases were bed ridden, and 72 (42.6%) could walk without help or with a cane. Non-survived patients had significantly larger intra-ventricular hemorrhage (IVH) (p < 0.001) and hematoma (p < 0.001) volume, higher serum glucose (p < 0.001) and blood pressure (p = 0.028), higher frequency of IVH (p < 0.001), and higher WBC count (p = 0.037). Sensitivity, specificity, positive predictive value (PPV), and negative predictive value (NPV) of SUSPEKT score at the 65 cut point were 82.97% (95% CI: 68.65% – 91.86%), 74.59% (95% CI: 65.76% – 81.84%), 55.71% (95% CI: 43.38% – 67.40%), and 91.91% (95% CI: 84.23% – 96.16%), respectively.

**Conclusion:**

Total accuracy of SUSPEKT score in predicting one-month mortality of non- traumatic ICH patients is in good range and it has 82% sensitivity and 92% NPV in this regard. It seems that we need further studies before applying the score in routine practice.

## Introduction

Intracranial hemorrhage (ICH) is accountable for 15% of all strokes but it is one of the most disabling forms of stroke (1). Spontaneous non-traumatic ICH is associated with high mortality rate worldwide (2). More than thirty percent of patients with ICH will not survive and only 20% of them will go on to be functional and live independently (3). The Global Burden of Disease 2010 Study showed a 47% increase in the absolute number of hemorrhagic strokes throughout the world during 1990-2010. The largest proportion of ICH incident cases (80%) and deaths (63%) occurred in low- and middle-income countries such as Sub-Saharan Africa, Central Asia and Southeast Asia (4). Mortality of ICH is estimated as forty percent in one month and 54% in one year (5). 

Therefore, outcome prediction of these patients is the area of interest for in charge physicians as well as patients and their associates. Predicting stroke outcomes is widely studied, and a lot of factors such as age, Glasgow coma scale, hematoma volume and location, and presence of intra-ventricular hemorrhage (IVH) are introduced and discussed as the risk factors of mortality in these patients (6-8). 

Despite previous investigations, there is no validated clinical scoring system for wide use in predicting the prognosis of ICH patients. In a recent study, SUSPEKT score was proposed as a simple, cheap and reproducible scoring system for 30-day clinical outcome prognosis in ICH patients. This score consists of six parameters: serum glucose, total hematoma volume, systolic blood pressure, existence of intra-ventricular hemorrhage, serum potassium level, and age (9). 

This study aimed to evaluate the accuracy of the mentioned clinical rule in predicting one-month mortality of ICH patients referred to emergency department.

## Methods

***Study design and setting***

This prospective cross sectional study was conducted on patients with primary non-traumatic ICH admitted to Imam Hossein Hospital, Tehran, Iran, from February 2017 to January 2018. SUSPEKT score was measured for each patient and its accuracy in prediction of one-month mortality was calculated. The study protocol was approved by ethics committee of Shahid Beheshti University of Medical Sciences (ethics code: IR.SMBU.RETECH.REC.1395.295). An informed consent form was completed by participants or their legal guardian. Patients had the right to decline to continue in every stage the study. 

***Participants***

Hemorrhagic stroke patients over 18 years old who were admitted to emergency department within 24 hours after the stroke were included. Patients with unstable hemodynamic status, pregnancy/ breastfeeding, previous neurologic deficit, sub-arachnoid hemorrhage (SAH), infra-tentorial hemorrhage, arteriovenous malformations, and history of brain tumor, as well as those who underwent neurosurgical evacuation or drainage, and had incomplete data were excluded. 

All patients were transported to our emergency department within 24 hours of stroke onset. If this point of time could not be ascertained, we used the last time when the patient was known to be well.

***Data gathering***

A researcher-made checklist was completed for each patient, which consisted of age, gender, history of smoking and alcohol usage, as well as systolic and diastolic blood pressure at the time of admission to emergency department. We also recorded laboratory data of patients including serum potassium, hemoglobin, serum glucose, white blood cell (WBC) and platelet counts. A senior emergency medicine resident was responsible for data gathering under the direct supervision of an emergency medicine specialist. 

***Outcome***

The main outcome was one-month mortality. We also categorized patients in five outcome categories as dead, walking without help, walking with help, bedridden and wheel-chair dependent.

***Procedure***

 After providing critical care and monitoring, all patients underwent brain computed tomography (CT) scan without contrast within 30 minutes of arrival, and if ICH was confirmed via CT scan the patient was evaluated for eligibility. Two consultant radiologists, who were blinded to the outcome, performed image analysis, independently. Brain CT scans were performed on 16-slice multi-detector CT scanners. Slice thickness was 5 to 10 mms for supra-tentorial and 2.5 to 4 mms for infra-tentorial regions. Images were transferred to an offline image processing workstation as DICOM (Digital Imaging and Communications in Medicine) files. Radiologists separated intracranial space from the skull and non-brain structures for analysis. The following variables were evaluated: total intracranial volume; total hematoma volume; intra-parenchymal hematoma volume; and intra-ventricular hematoma volume, each expressed as cm^3^. Additionally, relative volumes were defined as the ratio of total, intra-parenchymal, and intra-ventricular hematoma volumes to intra-cranial volume yielding variables (without unit).

***SUSPEKT score***

We calculated SUSPEKT score according to the previously published article by Rita Szepesi and colleagues in 2014 (9). This score is developed to predict the 30-day mortality of ICH patients using 6 factors including serum glucose, total hematoma volume, systolic blood pressure, presence of IVH, serum potassium level, and age. 

***Statistical analysis***

The sample size, considering α = 0.05, standard deviation = 3.5, d = 7.4, and power = 80%, was calculated to be 170 cases. The data were analyzed using SPSS version 21 statistical software. Mean ± standard deviation or frequency and percent were used for descriptive statistics. For comparison of the findings between groups, we used T-Test and Chi square test. The screening performance characteristics (sensitivity, specificity, positive predictive value (PPV), negative predictive value (NPV), positive likelihood ratio (PLR), and negative likelihood ratio (NLR)) of SUSPEKT score in prediction of one-month mortality was calculated using VassarStats medical calculator with 95% confidence intervals (CI). Total accuracy and the best cut point of SUSPEKT in this regard were calculated using the area under the receiver operating characteristic (ROC) curve. Accuracy of 0.90-0.100 was considered as excellent, 0.80-0.90 as good, 0.70-0.80 as fair, 0.60-0.70 as poor, and 0.50-.60 as fail. P value less than 0.05 was considered significant. 

**Table1:** Baseline characteristics of participants

**Variable **	**Value**
**Age (year)**	
20-39	13 (7.7)
40-59	49 (29.0)
>60	107 (63.3)
**Gender**	
Male	96 (56.8)
Female	73 (43.2
**History**	
Smoking	19 (11.2)
Alcohol abuse	75 (44.4)
**Blood pressure (mmHg)**	
Systolic	159.10 ± 34.95
Diastolic	80.22 ± 16.98
**Blood glucose level (mmol/L)**	
Mean ± SD	8.80± 4.29
**Laboratory parameters**	
Serum Potassium (meq/L)	3.99 ± 0.53
Hemoglobin (mg/dL)	13.24± 1.90
Platelet count (10^9^cells /liter)	234.18± 74.95
WBC count (10^9^cells/liter)	9.60± 3.21
**IVH size (cm** ^3^ **)**	
Mean ± SD	0.010± 0.026
**Total hematoma size (cm** ^3^ **)**	
Mean ± SD	0.039± 0.048

Data are presented as mean ± standard deviation (SD) or frequency (%). WBC: white blood cell; IVH: intraventricular hematoma.

**Table2:** Comparing the baseline characteristics of survived and non-survived cases

**Variable **	**Survived (n=122)**	**Dead (n= 47)**	**P**
**Gender **			
Female	52 (42.6)	21 (44.7)	0.47
Male	70 (57.4)	26 (55.3)	
**Age (year) **			
Mean± SD	62.04 ± 15.60	65.83 ± 14.78	0.81
**Serum potassium (meq/L)**			
Mean± SD	4.00 ± 15.60	3.95 ± 0.48	0.74
**Bleeding size (cm** ^3^ **)**			
IVH size	0.007 ± 0.017	0.026 ± 0.036	<0.001
Total hematoma	0.027 ± 0.037	0.069 ± 0.0589	<0.001
**IVH presence**			
Yes	19 (35.8)	34 (64.2)	<0.001
No	103 (88.8)	13 (11.2)	
**Serum glucose (mmol/L)**			
Mean± SD	7.9 ± 2.7	11.1 ± 6.2	<0.001
**Blood pressure (mmHg)**			
Systolic	155.4 ±31.2	168.5 ± 42.1	0.028
Diastolic	77.9 ± 15.8	86.1 ± 18.5	0.005
**Cell blood counts**			
WBC (10^9 ^cells/L)	9.2 ± 2.8	10.4 ± 3.9	0.037
Platelet (10^9 ^cells/L)	235.3 ± 63.8	231.2 ± 98.9	0.754
Hemoglobin (mg/dl)	13.3 ± 1.8	12.8 ± 2.0	0.131

Data are presented as mean ± standard deviation (SD) or frequency (%). IVH: intraventricular hematoma; WBC: white blood cell.

**Figure1:**
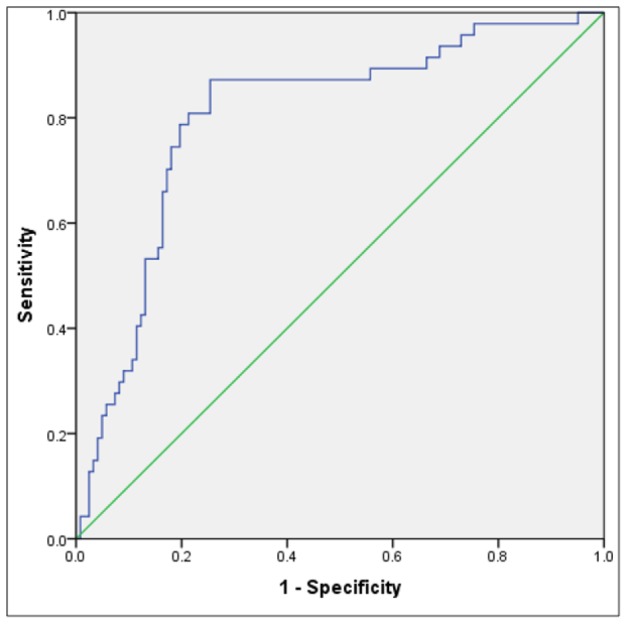
Area under the receiver operating characteristic (ROC) curve of SUSPEKT score in predicting one-month mortality of patients with intracranial hemorrhage

## Results

***Baseline characteristics of participants***

169 cases with the mean age of 63.09 ± 15.45 (21 – 96) years were studied (56.8% male). The baseline characteristics of patients are listed in table1. More than 90% of cases were in > 40 years range. The mean IVH and total hematoma size in this series were 0.010± 0.026 cm^3^ and 0.039± 0.048 cm^3^, respectively. After one month follow up 47 (27.8%) cases had died, 30 (17.7%) cases were bed ridden, and 72 (42.6%) could walk without help or with a cane. Table 2 compares the baseline characteristics of survived and non-survived cases. Non-survived patients had significantly larger IVH (p < 0.001) and hematoma (p < 0.001) volume, higher blood glucose level (p < 0.001) and blood pressure (p = 0.028), and higher WBC count (p = 0.037). 72.3% (34 cases) of non-survived patients had IVH, while only 15.6% (19 cases) of survived cases had simultaneous ventricular hemorrhage (p < 0.001).

***Accuracy of SUSPEKT score***

The mean SUSPEKT score of non-survived patients was significantly higher than survived cases (78.51 ± 22.64 vs 48.85 ± 28.3; p < 0.0001). The area under the ROC curve of SUSPEKT score in prediction of one-month mortality of ICH patients was 0.803 (95% CI: 0.727-0.879; P<0.001). The best cut point for the score in this regard was 65 (based on ROC curve). Sensitivity, specificity, PPV, NPV, PLR, and NLR of SUSPEKT score at the 65 cut point were 82.97% (95% CI: 68.65% – 91.86%), 74.59% (95% CI: 65.76% – 81.84%), 55.71% (95% CI: 43.38% – 67.40%), 91.91% (95% CI: 84.23% – 96.16%), 1.25 (95% CI: 0.89 – 1.75), and 0.09 (95% CI: 0.05 – 0.17), respectively.

## Discussion

Based on the findings of the present study, the total accuracy of SUSPEKT score in predicting one-month mortality of ICH patients is in good range and it has 82% sensitivity and 92% NPV in this regard. 

Despite wide researches performed on predicting mortality of primary ICH cases, there is no reliable and widely used scoring system in this regard (6, 10, 11). There are different prognostic models in predicting mortality of ICH (12, 13). These studies have been focused on age, low level of consciousness, location or volume of hemorrhage, existence of hydrocephalus in CT scan, and focal neurologic symptoms on admission as possible prognostic factors of outcome (14-17). 

Tsikriki et al., in their study regarding the prognostic factors of mortality following ICH, demonstrated the independent association of Apache II score, Sofa score, GCS on admission, and ICH volume with 30-day mortality (18).

In line with Szepesi et al. study, we showed that each item of SUSPEKT score, except for two variables of age and serum potassium level, significantly correlated with the 30-day outcome of ICH patients, independently (9). 

It is shown that serum glucose level is significantly associated with mortality in ICH patients (19, 20). Some other investigations showed that hypoglycemia is not a strong predictor for mortality (13, 21-23). It is also demonstrated that, acute hyperglycemia in patients with stroke, can be reflective of adverse findings in MRI and stereoscopy studies (23). In the present study, non-survived patients had significantly higher serum glucose level. 

In line with the findings of Taha Nisa, et al. and in disagreement with SUSPEKT score, there was no significant association between the mean age of patients and mortality (24). 

It is shown that volume of ICH can significantly predict 30-day mortality (11, 25). In addition, existence and size of IVH, is significantly associated with final outcome (26, 27). It is shown that increased hemorrhage predisposes patients to die within 4 weeks after stroke (28). Based on our findings, both IVH and total hematoma size were significantly higher in non-survived patients. 

Regarding the association of serum potassium level and mortality, the finding of this study was in disagreement with the findings of the SUSPEKT score derivation study.

The SUSPEKT score is difficult to calculate and based on our findings it seems that we need further comprehensive research before considering the SUSPEKT score as a screening test in our routine practice. The weight of some variables, such as serum potassium and age, in predicting mortality should be reevaluated and some factors such as the location of hemorrhage, cause of hemorrhage, and etc. should be more considered in future studies. Performing large-scale and multi-centric studies for assessing the value of SUSPEKT score can be helpful for evaluating the generalizability of findings.

## Limitations

Small sample size and focusing on the patients of one center were among the limitations of the present study. 

## Conclusion:

Based on the findings of the present study, the total accuracy of SUSPEKT score in predicting one-month mortality of ICH patients is in good range and it has 82% sensitivity and 92% NPV in this regard. 

## References

[1] Qureshi AI, Mohammad YM, Yahia AM, Suarez JI, Siddiqui AM, Kirmani JF (2005). A prospective multicenter study to evaluate the feasibility and safety of aggressive antihypertensive treatment in patients with acute intracerebral hemorrhage. Journal of intensive care medicine.

[2] Steiner T, Al‐Shahi Salman R, Beer R, Christensen H, Cordonnier C, Csiba L (2014). E uropean S troke O rganisation (ESO) guidelines for the management of spontaneous intracerebral hemorrhage. International journal of stroke.

[3] Sahni R, Weinberger J (2007). Management of intracerebral hemorrhage. Vascular health and risk management.

[4] Krishnamurthi RV, Moran AE, Forouzanfar MH, Bennett DA, Mensah GA, Lawes CM (2014). The global burden of hemorrhagic stroke: a summary of findings from the GBD 2010 study. Global heart.

[5] van Asch CJ, Luitse MJ, Rinkel GJ, van der Tweel I, Algra A, Klijn CJ (2010). Incidence, case fatality, and functional outcome of intracerebral haemorrhage over time, according to age, sex, and ethnic origin: a systematic review and meta-analysis. The Lancet Neurology.

[6] Ruiz-Sandoval JL, Chiquete E, Romero-Vargas S, Padilla-Martínez JJ, González-Cornejo S (2007). Grading scale for prediction of outcome in primary intracerebral hemorrhages. Stroke.

[7] Ziai WC, Carhuapoma JR (2018). Intracerebral Hemorrhage. Continuum: Lifelong Learning in Neurology.

[8] Sandeep YS, Guru MR, Jena RK, Kumar VAK, Agrawal A (2017). Clinical study to assess the outcome in surgically managed patients of spontaneous intracerebral hemorrhage. International journal of critical illness and injury science.

[9] Szepesi R, Széll IK, Hortobágyi T, Kardos L, Nagy K, Lánczi LI (2015). New prognostic score for the prediction of 30-day outcome in spontaneous supratentorial cerebral haemorrhage. BioMed research international.

[10] Broderick JP, Brott TG, Duldner JE, Tomsick T, Huster G (1993). Volume of intracerebral hemorrhage A powerful and easy-to-use predictor of 30-day mortality. Stroke.

[11] Weimar C, Benemann J, Diener H (2006). Development and validation of the Essen intracerebral haemorrhage score. Journal of Neurology, Neurosurgery & Psychiatry.

[12] Hemphill JC, Bonovich DC, Besmertis L, Manley GT, Johnston SC (2001). The ICH score. Stroke.

[13] Tuhrim S, Dambrosia JM, Price TR, Mohr JP, Wolf PA, Hier DB (1991). Intracerebral hemorrhage: external validation and extension of a model for prediction of 30‐day survival. Annals of Neurology: Official Journal of the American Neurological Association and the Child Neurology Society.

[14] Gomis M, Ois A, Rodriguez‐Campello A, Cuadrado‐Godia E, Jiménez‐Conde J, Subirana I (2010). Outcome of intracerebral haemorrhage patients pre‐treated with statins. European journal of neurology.

[15] Gebel Jr JM, Jauch EC, Brott TG, Khoury J, Sauerbeck L, Salisbury S (2002). Relative edema volume is a predictor of outcome in patients with hyperacute spontaneous intracerebral hemorrhage. Stroke.

[16] Nilsson OG, Lindgren A, Brandt L, Säveland H (2002). Prediction of death in patients with primary intracerebral hemorrhage: a prospective study of a defined population. Journal of neurosurgery.

[17] Reinhard M, Neunhoeffer F, Gerds TA, Niesen W-D, Buttler K-J, Timmer J (2010). Secondary decline of cerebral autoregulation is associated with worse outcome after intracerebral hemorrhage. Intensive care medicine.

[18] Tsikriki S, Karathanou A, Kokoris I, Topalis T, Voutsinas E, Antoniou A (2015). Prognostic factors of 30-days mortality in primary intracerebral hemorrhage.

[19] Fogelholm R, Murros K, Rissanen A, Avikainen S (2005). Admission blood glucose and short term survival in primary intracerebral haemorrhage: a population based study. Journal of Neurology, Neurosurgery & Psychiatry.

[20] Stead LG, Jain A, Bellolio MF, Odufuye A, Gilmore RM, Rabinstein A (2010). Emergency Department hyperglycemia as a predictor of early mortality and worse functional outcome after intracerebral hemorrhage. Neurocritical care.

[21] Hemphill III JC, Greenberg SM, Anderson CS, Becker K, Bendok BR, Cushman M (2015). Guidelines for the management of spontaneous intracerebral hemorrhage: a guideline for healthcare professionals from the American Heart Association/American Stroke Association. Stroke.

[22] Tetri S, Juvela S, Saloheimo P, Pyhtinen J, Hillbom M (2009). Hypertension and diabetes as predictors of early death after spontaneous intracerebral hemorrhage. Journal of neurosurgery.

[23] Lee S-H, Kim B, Bae H-J, Lee J, Lee J, Park B-J (2010). Effects of glucose level on early and long-term mortality after intracerebral haemorrhage: the Acute Brain Bleeding Analysis Study. Diabetologia.

[24] Nisar T, Alchaki A, Hillen MJCn, neurosurgery (2018). Validation of ICH score in a large urban population.

[25] Tsikriki S, Karathanou A, Kokoris I, Topalis T, Voutsinas E, Antoniou A (2015). Prognostic factors of 30-days mortality in primary intracerebral hemorrhage. Intensive care medicine experimental.

[26] Ueshima H, Reza Choudhury S, Okayama A, Hayakawa T, Kita Y, Kadowaki T (2004). Cigarette smoking as a risk factor for stroke death in Japan: NIPPON DATA80. Stroke.

[27] Wong K (1999). Risk factors for early death in acute ischemic stroke and intracerebral hemorrhage: a prospective hospital-based study in Asia. Stroke.

[28] Brott T, Broderick J, Kothari R, Barsan W, Tomsick T, Sauerbeck L (1997). Early hemorrhage growth in patients with intracerebral hemorrhage. Stroke.

